# Service utilisation trends in the manual therapy professions within the Australian private healthcare setting between 2008 and 2017

**DOI:** 10.1186/s12998-020-00338-1

**Published:** 2020-09-21

**Authors:** Reidar P. Lystad, Benjamin T. Brown, Michael S. Swain, Roger M. Engel

**Affiliations:** 1 Australian Institute of Health Innovation, Macquarie University, Sydney, Australia; 2 Department of Chiropractic, Macquarie University, Sydney, Australia

**Keywords:** Health services, Cost, Manual therapy, Chiropractic, Osteopathy, Physiotherapy

## Abstract

**Background:**

Better understanding of the dynamics and temporal changes in manual therapy service utilisation may assist with healthcare planning and resource allocation. The objectives of this study were to quantify, describe, and compare service utilisation trends in the manual therapy professions within the Australian private healthcare setting between 2008 and 2017.

**Methods:**

Data regarding the number of services, total cost, and benefits paid were extracted for each manual therapy profession (i.e. chiropractic, osteopathy, and physiotherapy) for the period 2008–2017 from the Australian Prudential Regulation Authority. The number of registered providers for each profession were obtained from the Australian Health Practitioner Regulation Agency. Descriptive statistics were produced for two time periods (i.e. 2008–2012 and 2013–2017) for each manual therapy profession. Annual percentage change during each time period was estimated by fitting Poisson regression models. Test for the equality of regression coefficients was used to compare the trends in the two time periods within each profession, and to compare the trends across professions within a time period.

**Results:**

A cumulative total of 198.6 million manual therapy services with a total cost of $12.8 billion was provided within the Australian private healthcare setting between 2008 and 2017. Although service utilisation and total cost increased throughout the ten-year period, the annual growth was significantly lower during 2013–2017 than 2008–2012. Whereas osteopathy and physiotherapy experienced significant annual growth in the number of services and total cost during 2013–2017, negative growth in the number of services was observed for chiropractic during the same period. The annual number of services per provider declined significantly for chiropractic and physiotherapy between 2013 and 2017.

**Conclusion:**

Service provision under private health insurance general treatment cover constitute a major source of revenue for manual therapy professions in Australia. Although manual therapy service utilisation increased throughout the ten-year period from 2008 to 2017, the annual growth declined. There were diverging trends across the three professions, including significantly greater decline in annual growth for chiropractic than for osteopathy and physiotherapy.

## Background

In Australia, healthcare services are delivered by both the public and private sectors [1]. In the 2017–2018 financial year, total healthcare expenditure in Australia amounted to $185.4 billion [2]. Government funding accounted for over two-thirds (68.3%) of this expenditure, with private health insurers contributing $16.6 billion (9.0%) of the total funding [2]. In addition to the net contributions, which predominantly originated from premium payments by members, private health insurers also administered $5.9 billion in government funding in the form of premium rebates [2]. Individuals and private health insurers spent $2.2 billion and $0.9 billion, respectively, on primary healthcare services provided by health professionals other than medical doctors and dentists (e.g. audiologists, chiropractors, osteopaths, physiotherapists, optometrists, podiatrists, practice nurses, and speech pathologists) [2]. This accounted for approximately 1.7% of the total national health expenditure in the 2017–2018 financial year.

Musculoskeletal (MSK) conditions represent a major public health burden. Low back and neck pain are leading causes of years lived with disability and their burden is growing [3]. In 2016, an estimated US$135 billion was spent on treatment services for low back and neck pain in the United States alone, of which approximately 57% was paid by private health insurance [4]. An estimated 3.2 million Australians experienced low back pain in 2017 [3]. This amounted to a total of 360,000 years lived with disability, which corresponded to an 18% increase in years lived with disability since 2007 [3]. The increasing burden of MSK conditions such as low back and neck pain is driven primarily by population ageing which is occurring rapidly worldwide [5, 6]. Because this demographic shift is enduring, the demand for healthcare services to manage MSK conditions is likely to increase in the coming decades.

Manual therapy providers (i.e. chiropractors, osteopaths, and physiotherapists) deliver a substantial proportion of healthcare services for treating and managing MSK conditions. In Australia, chiropractors, osteopaths, and physiotherapists are registered healthcare practitioners trained to diagnose, treat, and manage patients with MSK conditions. Manual therapy services are predominately paid for by non-government sources, primarily by individuals and private health insurers. The provision of manual therapy services is influenced by several internal and external factors. Internal factors include graduate capabilities, code of conduct, and self-regulated professional behaviour and practice, whereas external factors include government legislation, private health insurance policies, individual and community preferences, population demographics, economic circumstances (e.g. level of household disposable income), and health status of the population [7]. These factors are components of a complex system in which a change in one factor can influence other factors. To better understand the dynamics of changes in manual therapy service utilisation and to assist with healthcare planning and future resource allocation, it is necessary to quantify and describe recent trends in manual therapy service utilisation. Unfortunately, the most recent analysis of manual therapy service utilisation trends is more than five years old and does not include data beyond 2012 [8]. Therefore, the specific objectives of this study were: (1) to quantify and describe service utilisation trends in the manual therapy professions (i.e. chiropractic, osteopathy, and physiotherapy) within the Australia private healthcare setting between 2008 and 2017; (2) to compare service utilisation trends across manual therapy professions during 2008–2012 and 2013–2017; and (3) to compare service utilisation trends within each manual therapy profession during 2008–2012 and 2013–2017.

## Methods

### Data sources

**Fig. 1:**
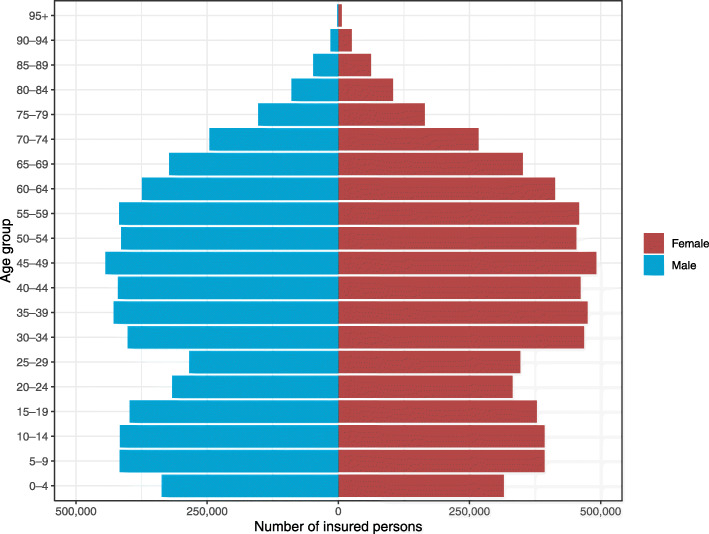
Number of insured persons by sex and age group in 2017

Quarterly reports and data cubes detailing private health insurance industry activity are available through the Australian Prudential Regulation Authority (APRA) website [9]. For this study, quarterly data regarding the number of services, total cost of services, and benefits paid for these services, were extracted for each manual therapy profession (i.e. chiropractic, osteopathy, and physiotherapy) for the period 2008–2017. Data were also obtained from the APRA website regarding the number of individuals with private health insurance (i.e. general treatment cover). For consistency, the number of individuals with general treatment cover were taken from the December quarter for each year during the period 2008–2017. Figure 1 shows the number insured persons by sex and age group in 2017.

Estimates of the resident population of Australia were obtained from demographic statistics provided by the Australian Bureau of Statistics (ABS) [10]. For consistency, the estimates of the resident population were taken from the December quarter for each year during the period 2008–2017.

The number of registered providers in each manual therapy profession were obtained from the Australian Health Practitioner Regulation Agency (AHPRA) website [11]. Registrant data is captured quarterly by each of the relevant boards [12–14]. Registrant data is available from 2012 onwards. For consistency, the number of registered providers were taken from the December publication of the registrant data for each year between 2012 and 2017. Registrants listed as ‘limited’ or ‘non-practicing’ were excluded from the sample. Providers working in the public sector were also omitted, as services rendered under this framework are not covered by private health insurers. The number of providers working in the private sector was estimated for each profession. Because chiropractic and osteopathy operate almost exclusively in the private sector, the number of providers working in the private sector was considered to be equal to the number of registrants. Physiotherapy, on the other hand, operates in both the private and public sectors. For the purposes of this study, the proportion of registered physiotherapists working in the private sector was estimated to be 63.5%, as per the 2012 National Health Workforce Report [15].

### Operational definitions

Private health insurers provide several types of cover: hospital treatment only, combined hospital and general treatment, and general treatment only. A policy that includes general treatment cover represents insurance for treatment that is intended to manage or prevent a disease, injury, or condition, and is separate to hospital treatment. That is, general treatment excludes services provided in hospitals, as well as services which fall under the banner of Chronic Disease Management (formerly Enhanced Primary Care). In the context of private health insurance data, a *service* represents one visit to a healthcare provider. The *total cost* of services represents the sum of the fees charged at the time of consultation (fee for service) to individuals who are insured under a policy that includes general treatment cover. A *benefit paid* is the portion of the total fee for a service that is reimbursed by a health insurer under general treatment cover. The portion of the total fee not reimbursed by a health insurer is considered an *out-of-pocket cost* to the individual.

### Data management and analysis

Quarterly data regarding the number of services, total cost of services, and benefits paid for these services were summated to produce yearly statistics for each manual therapy profession. Out-of-pocket cost of services was calculated by subtracting benefits paid for services from the total cost of services. All dollar values were adjusted for inflation using the Reserve Bank of Australia’s online inflation calculator and reported in 2017 Australian dollars [16]. The main outcome variables were number of services per year, total cost of services per year, benefits paid for services per year, and out-of-pocket cost of services per year. The number of services was used as numerator and the number of providers was used as denominator to calculate the number of services per provider per year. The proportion of individuals with general treatment cover per year was calculated using the number of individuals with general treatment cover as the numerator and the estimated resident Australian population as denominator for each year during the study period. Lastly, the number of individuals with general treatment cover was used as denominator to generate the following secondary outcome variables for additional sensitivity analyses: number of services per 100,000 insured population per year, total cost of services per 100,000 insured population per year, benefits paid for services per 100,000 insured population per year, and out-of-pocket cost of services per 100,000 insured population per year.

Descriptive statistics were calculated for each outcome variable during two time periods (i.e. 2008–2012 and 2013–2017) for each manual therapy profession. Overall percentage change during each time period was calculated for each outcome variable. Annual percentage change during each time period was estimated by fitting Poisson regression models for each outcome variable. For each outcome variable, a test for the equality of regression coefficients was used to compare the trends across the two time periods for each manual therapy profession, and to compare the trends across manual therapy professions within a single time period [17]. All statistical analyses were conducted using R, version 3.5.1 (R Foundation for Statistical Computing, Vienna, Austria).

## Results

During the ten-year period 2008–2017, the three manual therapy professions in Australia provided a total of 198.6 million services with a cumulative total cost of approximately $12.8 billion. Table 1 provides an overview of cumulative number of services, total cost, benefits paid, and out-of-pocket cost by manual therapy profession between 2008 and 2017. Physiotherapy and chiropractic accounted for 49.8% and 46.4% of services and 53.3% and 41.7% of the cumulative total cost, respectively, while osteopathy accounted for 3.9% of services and 5.0% of the cumulative total cost.

**Table 1:** Cumulative number of services, total cost, benefits paid, and out-of-pocket cost by manual therapy profession in Australia during 2008–2017

	Chiropractic	Osteopathy	Physiotherapy	Total
**2008–2017**				
Number of services	92,080,447	7,657,460	98,846,368	198,584,275
Total cost	$5,317,361,571	$641,468,929	$6,806,953,283	$12,765,783,784
Benefits paid	$2,747,206,748	$271,923,007	$3,433,029,328	$6,452,159,084
Out-of-pocket cost	$2,570,154,823	$369,545,922	$3,373,923,955	$6,313,624,700

Table 2 provides an overview of the mean annual number of services, total cost, benefits paid, and out-of-pocket cost by manual therapy profession during 2008–2012 and 2013–2017. For the three manual therapy professions combined, the mean annual number of services and total cost increased from 18.1 million and $1.1 billion during 2008–2012 to 21.6 million and $1.4 billion during 2012–2017.

**Table 2:** Mean annual number of services, total cost, benefits paid, and out-of-pocket cost by manual therapy profession in Australia during 2008–2012 and 2013–2017

	Chiropractic	Osteopathy	Physiotherapy	Total
**2008–2012**				
Number of services	8,843,123	608,661	8,688,070	18,139,854
Total cost	$499,262,656	$50,005,462	$574,407,353	$1,123,675,471
Benefits paid	$254,685,115	$22,399,268	$290,222,043	$567,306,426
Out-of-pocket cost	$244,577,541	$27,606,195	$284,185,310	$556,369,045
**2013–2017**				
Number of services	9,572,967	922,831	11,081,203	21,577,001
Total cost	$564,209,658	$78,288,323	$786,983,304	$1,429,481,285
Benefits paid	$294,756,234	$31,985,334	$396,383,823	$723,125,391
Out-of-pocket cost	$269,453,424	$46,302,990	$390,599,481	$706,355,895

**Fig. 2:**
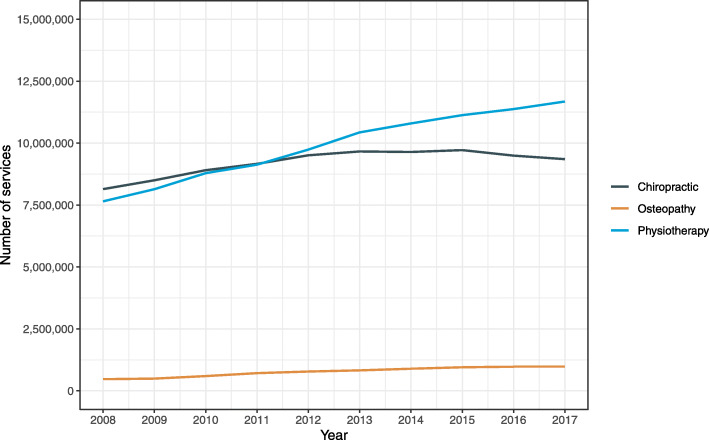
Annual number of services by manual therapy profession in Australia during 2008–2017

**Fig. 3:**
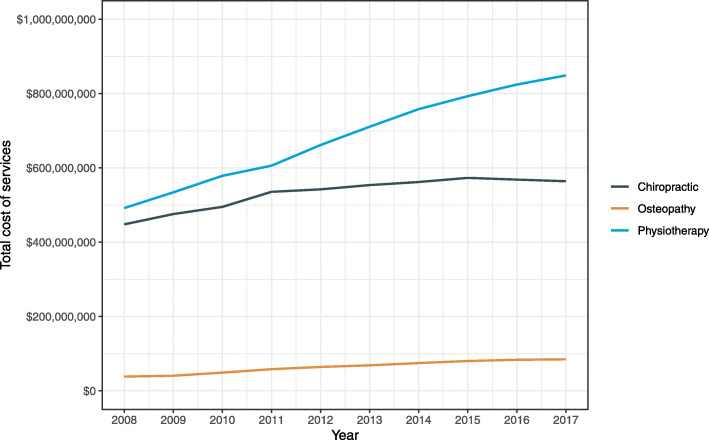
Annual total cost of services by manual therapy profession in Australia during 2008–2017

The annual number of services and total cost of services during 2008–2017 for each manual therapy profession are shown in Figs. 2 and 3, respectively. During the ten-year period, the largest overall growth was observed for osteopathy with the annual number of services and total cost increasing by 108.7% and 121.6%, respectively. For physiotherapy, the annual number of services and total cost increased by 52.7% and 72.6%, respectively. The lowest overall growth was observed for chiropractic with the annual number of services and total cost increasing by 14.9% and 25.9%, respectively.

Table 3 provides an overview of the annual percent change of number of services, total cost, benefits paid, and out-of-pocket cost by manual therapy profession 2008–2012 and 2013–2017. The annual growth in number of services and total cost was significantly lower during 2013–2017 versus 2008–2012 for all three manual therapy professions. All three professions experienced significant annual growth in the number of services and total cost during 2008–2012. However, the observed annual growth in the number of services was significantly higher for osteopathy (13.9% [95%CI: 13.8%, 14.0%]) than for chiropractic (3.8% [95%CI: 3.8%, 3.9%]; *p* < 0.001) and physiotherapy (6.0% [95%CI: 5.9%, 6.0%]; p < 0.001). Similarly, the observed annual growth in the total cost was significantly higher for osteopathy (14.0% [95%CI: 14.0%, 14.0%]) than for chiropractic (5.0% [95%CI: 5.0%, 5.0%]; p < 0.001) and physiotherapy (7.2% [95%CI: 7.2%, 7.2%]; p < 0.001). Whereas osteopathy and physiotherapy experienced significant annual growth in the number of services and total cost during 2013–2017, negative growth in the number of services was observed for chiropractic during this period. The annual percent change in the number of services was significantly lower for chiropractic (− 0.8% [95%CI: − 0.8%, − 0.8%]) than for osteopathy (4.2% [95%CI: 4.2%, 4.3%]; *p* < 0.001) and physiotherapy (2.8% [95%CI: 2.8%, 2.8%]; p < 0.001). Similarly, the annual percent change in the total cost was significantly lower for chiropractic (0.5% [95%CI: 0.5%, 0.5%]) than for osteopathy (5.3% [95%CI: 5.3%, 5.3%]; *p* < 0.001) and physiotherapy (4.4% [95%CI: 4.4%, 4.4%]; p < 0.001).

**Table 3:** Annual percent change with 95% confidence interval of number of services, total cost, benefits paid, and out-of-pocket cost by manual therapy profession in Australia during 2008–2012 and 2013–2017

	Chiropractic	Osteopathy	Physiotherapy
**2008–2012**			
Number of services	3.8% (3.8%, 3.9%)*	13.9% (13.8%, 14.0%)*	6.0% (5.9%, 6.0%)*
Total cost	5.0% (5.0%, 5.0%)*	14.0% (14.0%, 14.0%)*	7.2% (7.2%, 7.2%)*
Benefits paid	4.8% (4.8%, 4.8%)*	11.8% (11.8%, 11.8%)*	6.8% (6.8%, 6.8%)*
Out-of-pocket cost	5.1% (5.1%, 5.1%)*	15.8% (15.8%, 15.9%)*	7.6% (7.6%, 7.6%)*
**2013–2017**			
Number of services	−0.8% (− 0.8%, − 0.8%)*	4.2% (4.2%, 4.3%)*	2.8% (2.8%, 2.8%)*
Total cost	0.5% (0.5%, 0.5%)*	5.3% (5.3%, 5.3%)*	4.4% (4.4%, 4.4%)*
Benefits paid	− 0.3% (− 0.3%, − 0.3%)*	3.2% (3.2%, 3.2%)*	3.4% (3.4%, 3.4%)*
Out-of-pocket cost	1.3% (1.3%, 1.3%)*	6.7% (6.7%, 6.7%)*	5.4% (5.4%, 5.4%)*

* *p* < 0.001

**Fig. 4:**
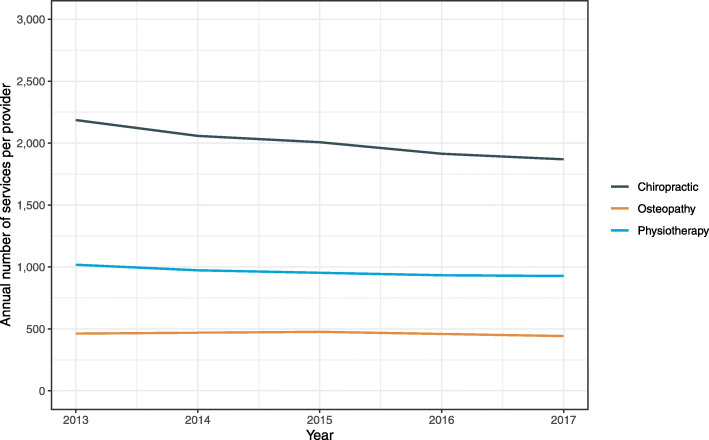
Annual number of services per provider by manual therapy profession in Australia during 2013–2017

The number of providers increased significantly for all three manual therapy professions during 2013–2017. The annual percent change was 5.4% (95%CI: 4.0%, 6.7%; *p* < 0.001) for osteopathy, 5.0% (95%CI: 4.5%, 5.5%; p < 0.001) for physiotherapy, and 3.0% (95%CI: 2.1%, 3.9%; p < 0.001) for chiropractic. The annual percent increase in number of providers was significantly greater in osteopathy (*p* = 0.005) and physiotherapy (p < 0.001), compared to chiropractic. The mean annual number of services per provider during 2013–2017 was 2007 for chiropractic, 961 for physiotherapy, and 462 for osteopathy. The annual number of services per provider during 2013–2017 decreased for all three manual therapy professions (Fig. 4). The observed decline in annual number of services per provider was statistically significant for chiropractic (− 3.9% [95%CI: − 5.3%, − 2.5%]; *p* < 0.001) and physiotherapy (− 2.3% [95%CI: − 4.3%, − 0.3%]; *p* = 0.024), but not for osteopathy (− 1.1% [95%CI: − 4.0%, 1.8%]; *p* = 0.462).

Although the number of individuals with general treatment cover increased throughout the ten-year study period, the annual growth in number of individuals with general treatment cover deviated from the general population growth. While the mean annual growth of the Australian population was 1.6% and 1.5% during 2008–2012 and 2013–2017, respectively, the mean annual growth in number of individuals with general treatment cover declined from 3.1% during 2008–2012 to 1.3% during 2013–2017. In other words, the proportion of the population with general treatment cover increased from 51.4% in 2008 to a peak of 55.7% in 2015, before declining to 54.6% in 2017. Because the number and proportion of individuals with general treatment cover varied from year to year, additional trend analyses were conducted using secondary outcome variables. The results from these analyses are provided in Table 4.

**Table 4:** Annual percent change with 95% confidence interval of number of services per 100,000 insured population, total cost per 100,000 insured population, benefits paid per 100,000 insured population, and out-of-pocket cost per 100,000 insured population by manual therapy profession in Australia during 2008–2012 and 2013–2017

	Chiropractic	Osteopathy	Physiotherapy
**2008–2012**			
Number of services	0.8% (0.7%, 0.8%)*	10.8% (10.7%, 10.9%)*	2.9% (2.9%, 2.9%)*
Total cost	1.9% (1.9%, 1.9%)*	11.0% (10.9%, 11.0%)*	4.1% (4.1%, 4.1%)*
Benefits paid	1.7% (1.7%, 4.8%)*	8.7% (8.7%, 8.7%)*	3.7% (3.7%, 3.7%)*
Out-of-pocket cost	2.1% (2.1%, 2.1%)*	12.8% (12.8%, 1.9%)*	4.5% (4.5%, 4.5%)*
**2013–2017**			
Number of services	−2.1% (−2.2%, −2.1%)*	2.9% (2.8%, 3.0%)*	1.4% (1.4%, 1.5%)*
Total cost	−0.9% (− 0.9%, − 0.9%)*	4.0% (3.9%, 4.0%)*	3.0% (3.0%, 3.0%)*
Benefits paid	−1.6% (−1.6%, −1.6%)*	1.9% (1.8%, 1.9%)*	2.0% (2.0%, 2.0%)*
Out-of-pocket cost	0.0% (0.0%, 0.0%)*	5.4% (5.4%, 5.4%)*	4.1% (4.1%, 4.1%)*

* *p* < 0.001

## Discussion

The three manual therapy professions provided nearly 200 million healthcare services at a total cost of approximately $12.8 billion under Australian private health insurance cover in the period from 2008 to 2017. Physiotherapy accounted for the largest annual total cost throughout the ten-year period, and surpassed chiropractic in 2011 as the profession delivering the most services per year. The annual number and total cost of services increased during the ten-year period for the three professions, with more pronounced growth in 2008–2012 than in 2013–2017. Growth in service utilisation varied by profession, with osteopathy experiencing the largest growth in the annual number and total cost of services during the ten-year period. Unlike osteopathy and physiotherapy, chiropractic had a negative growth in the annual number of services provided between 2013 and 2017. Although the number of providers increased for all three professions during 2013–2017, the annual number of services per provider declined. The decline was particularly pronounced for chiropractic; however, the annual number of services per chiropractor remained two and four times greater than for physiotherapists and osteopaths, respectively.

In Australia, manual therapy services are predominately paid for by non-government sources, primarily by individuals and private health insurers. Individual and private health insurer spending on primary healthcare services by health professionals other than medical doctors and dentists accounted for approximately 1.7% of the total national health expenditure in the 2017–2018 financial year [2]. A substantial proportion of this expenditure would have been for manual therapy services. Our data shows that the total cost of manual therapy services billed through private health insurers, which excludes services paid in full by individuals, was $1.5 billion in 2017. This represents approximately 0.8% of the annual national health expenditure. It is worth noting that manual therapy services billed through private health insurers represent approximately 80% of the total revenue for chiropractic and osteopathy and 50% for physiotherapy [7, 18]. This suggests that chiropractic and osteopathy are more reliant on revenue derived from private health insurers than physiotherapy, which may leave the two professions more exposed to changes in the private health insurance sector.

Demand for manual therapy services is influenced by disposable household income, both directly via individual spending and indirectly through the uptake of private health insurance with general treatment cover. The median weekly disposable household income in Australia increased by approximately 4.5% during the ten-year study period, with slower growth in the most recent years [19]. This corresponds well with our data that showed the proportion of the population with general treatment cover increased from 2008 to 2015, before declining during the most recent years. Thus, it appears that the overall manual therapy service utilisation trends observed in this study could be explained, at least in part, by changes in disposable household income.

Another important external influence on demand for manual therapy services is changes in population health characteristics. For instance, an increasing incidence of hospitalisations for road traffic trauma, falls, and sports-related injury may have influenced the demand for injury rehabilitation services [2, 20, 21]. Similarly, an ageing population and an increase in community health awareness may have influenced the demand for geriatric and preventative healthcare services [22–24]. Although such changes in population health characteristics increase the need for manual therapy services, the changes in demand could vary considerably across the three manual therapy professions. Consumer choice may depend on perceived differences in the three professions’ scope of practice, level of expertise, specific types of therapeutic modalities, and degree of integration with the broader healthcare system. In particular, consumer choice may be influenced by established referral pathways for particular types of healthcare services (e.g. fall prevention, post-injury rehabilitation, neurological rehabilitation, and pulmonary rehabilitation) [25, 26]. For instance, among a cohort of individuals with a transport-related whiplash injury in Victoria, Australia, between 2000 and 2013, there were more than three times as many compensation payments for physiotherapy services than for chiropractic services [27].

Fee structures may also influence the demand for services [7]. Benefits paid by private health insurers represents a proportion of the total cost of services, with the remaining proportion being the out-of-pocket cost to the patient. Firstly, the proportion of benefits paid declined for all three professions from 2008–2012 to 2013–2017 (i.e. chiropractic: from 53% to 51%; osteopathy: from 43% to 39%; physiotherapy: from 51% to 49%). This suggests that the total cost of manual therapy services is increasing at a greater rate than what private health insurers are willing to compensate their members in terms of benefits paid. Secondly, whereas osteopathy and physiotherapy experienced relatively strong growth in annual benefits paid and out-of-pocket cost during the most recent time period (i.e. 2013–2017), chiropractic appeared to have plateaued. Lastly, our data revealed notable differences in the relative proportions of benefits paid versus out-of-pocket cost for the three professions (i.e. chiropractic: 52% versus 48%; osteopathy: 41% versus 59%; physiotherapy: 50% versus 50%). Further research is needed to elucidate to what extent disparities in benefits paid for manual therapy services influence consumer choice and whether that causes diverging trends in service utilisation across the three professions.

Industry awareness and acceptance is a potential driver of consumer choice that is influenced by internal factors, and this may explain the plateau in the annual number of chiropractic services observed between 2013 and 2017. In Australia, instances of unprofessional and unethical attitudes and actions of individual chiropractors have generated a string of negative media coverage that have resulted in reputational damage for the chiropractic profession. For example, there are chiropractors who have promoted anti-vaccination views to their patients [28], entered public hospital maternity wards and treated newborns without authorisation [29], and made unsupported claims of benefit in their advertising material [30]. In addition, medical specialists have expressed concerns over the safety of aspects of chiropractic practice [31]. In response, the chiropractic regulator in Australia (i.e. the Chiropractic Board of Australia) was compelled to release three position statements on these matters: one related to advertising [32], one on the provision of health information [33], and one on paediatric care [34]. Having a strong and reputable industry body raises the awareness and acceptance of an industry and mitigates reputational damage. The chiropractic profession in Australia is represented by two industry bodies, both of which underwent rebranding between 2015 and 2018. This rebranding may itself have been a reaction to the increased public scrutiny and changing reputational standing of the chiropractic profession. Finally, relationships with other health professionals in the marketplace are important for industry success. In this context, strong professional relationships with general practitioners are important because they function as gatekeepers to all government and some private health insurance schemes. Whereas general practitioner referrals to physiotherapists increased significantly from 2009 to 2015 [35], a survey of general practitioners conducted in 2014 found less favourable professional attitudes and growing intolerance towards chiropractors and osteopaths [36, 37].

It is important to consider the supply side when interpreting the observed service utilisation trends presented herein. In the present report, we were able to include data on the annual growth in number of providers during the most recent five-year period (i.e. 2013–2017). These data show that the three professions experienced significant growth in the number of providers, while the average number of services per provider declined. However, the decline in the annual number of services per provider was far more pronounced for chiropractic than for physiotherapy and osteopathy. It is important to point out that the annual number of services per chiropractor remained two and four times greater than for physiotherapists and osteopaths, respectively. The reasons for the steeper decline in chiropractic is unclear, and there may multiple explanations. For instance, it is possible that the chiropractic profession in Australia has reached a saturation point in the number of providers. This would mirror observations from North America that suggest the chiropractic profession has been in oversupply since the turn of the century [38, 39]. Alternatively, perhaps the phenomenon of high-volume, low-value service provision, which subsists among a subset of the chiropractic profession [40–42], is becoming less sustainable as higher value models of care become more available external to the chiropractic profession [43, 44].

### Strengths and limitations

This research builds on our previous report of manual therapy service utilisation in Australia based on private health insurance data from 1998 to 2012 [8]. In our previous study we observed what appeared to be slower growth in service utilisation in chiropractic relative to physiotherapy and osteopathy from about 2006 onward; however, we did not conduct any formal statistical comparisons. In the present research, we extended our analysis of manual therapy service utilisation by estimating the number of services, total costs, and benefits paid for the ten-year period between 2008 and 2017. We added an inflation adjustment for all dollar values to remove the effect of inflation from our analyses. More importantly, we applied more sophisticated analytical techniques to formally quantify and compare trends across professions and time periods (i.e. 2008–2012 and 2013–2017). Not only have we quantified our previous observation regarding slower growth in service utilisation in chiropractic during 2008–2012 [8], we have also demonstrated that the growth slowed even further during 2013–2017.

Public reporting of registrant and workforce data by AHPRA has improved since 2012, which allowed us to include analyses of annual services per provider for the most recent five-year period (i.e. 2013–2017). We believe this strengthens and adds value to the present report. In an attempt to account for fluctuations in the number of people with private health insurance, we provided supplementary analyses in which the outcome variables were standardised using per 100,000 persons with private health insurance as the denominator. These supplementary analyses generated similar findings in terms of differences in trends across professions, although the magnitude of annual growth estimates was tempered by 3.1% and 1.3% for the two time periods (i.e. 2008–2012 and 2013–2017), respectively. These figures correspond to the annual percentage change in the number of people with private health insurance during the two time periods. Lastly, because our study was limited to private health insurance data, the estimates and comparisons of trends presented herein can only be generalised to services provided under private health insurance general treatment cover.

## Conclusion

Service provision under private health insurance general treatment cover constitute a major source of revenue for manual therapy professions in Australia. Although manual therapy service utilisation increased throughout the ten-year period from 2008 to 2017, the annual growth declined. There were diverging trends across the three professions, including significantly greater decline in annual growth for chiropractic than for osteopathy and physiotherapy. Future research is needed to determine the extent to which external and internal factors have contributed to the diverging trends in service utilisation across the three manual therapy professions in Australia during this period, and to investigate whether the trends have persisted beyond 2017.

## Data Availability

The dataset generated and analysed during this research is available in the figshare repository: 10.6084/m9.figshare.12178845.

## Abbreviations

ABS: Australian Bureau of Statistics
AHPRA: Australian Health Practitioner Regulation Agency
APRA: Australian Prudential Regulation Authority
MSK: Musculoskeletal
